# Age‐ and sex‐associated variability in lamotrigine prescription patterns and clearance

**DOI:** 10.1111/epi.70028

**Published:** 2026-02-02

**Authors:** Charul Avachat, Yuhan Long, Ashley Petersen, Angela K. Birnbaum, Sima I. Patel

**Affiliations:** ^1^ Experimental & Clinical Pharmacology University of Minnesota Minneapolis Minnesota USA; ^2^ Division of Biostatistics and Health Data Science University of Minnesota Minneapolis Minnesota USA; ^3^ Department of Neurology University of Minnesota Minneapolis Minnesota USA; ^4^ Present address: Bristol Myers Squibb Princeton New Jersey USA

**Keywords:** age, lamotrigine, menopause, pharmacokinetics, sex

## Abstract

**Objective:**

Lamotrigine is one of the most widely prescribed antiseizure medication (ASM) and mood stabilizer in the United States due to its favorable side‐effect profile, lower risk of teratogenicity, and minimal drug–drug interactions. This study aimed to examine age‐ and sex‐associated variability in prescribing and pharmacokinetics, focusing on postmenopausal women.

**Methods:**

Data were from electronic health records. Individuals were included if ≥18 years and received an ASM between January 1, 2015 and December 31, 2021. Lamotrigine prescriptions were compared based on age, sex, epilepsy diagnosis, and monotherapy/polytherapy. Statistical comparisons of proportions were conducted using two‐proportion tests. To characterize age‐ and sex‐related differences in LTG apparent oral clearance and assess the impact of covariates, linear mixed‐effects modeling was employed.

**Results:**

Records were available for 314 890 individuals, with 23 906 patients being prescribed lamotrigine at least once (as monotherapy or polytherapy) for both epilepsy and non‐epilepsy diagnoses. The lamotrigine prescription rate was lower in postmenopausal women compared to younger women but higher than in older men, irrespective of diagnosis. Notably, lamotrigine was prescribed as monotherapy more frequently to patients without epilepsy than those with epilepsy, regardless of sex and age. The clearance of lamotrigine was 22% lower in postmenopausal women compared to younger women and 9% in older men. Lamotrigine clearance increased by 49% and 11% with co‐administration of inducers or the presence of smoking, respectively. Lamotrigine clearance decreased by 51% in the presence of an inhibiting medication.

**Significance:**

Prescription rates for lamotrigine varied between patients with epilepsy and those with non‐epilepsy conditions. Age and sex differences in pharmacokinetics suggest the need for lamotrigine dose adjustments, highlighting the importance of therapeutic drug monitoring in personalized epilepsy care. Lamotrigine use was less frequent in postmenopausal women compared to younger women but higher compared to older men. Postmenopausal women were prescribed lamotrigine as monotherapy to a lesser extent than younger women and older men.

**Key points**

- The lamotrigine (LTG) prescription rate was lower in postmenopausal women compared to younger women but higher than in older men, irrespective of diagnosis.
- LTG was prescribed as monotherapy more frequently to patients without epilepsy than those with epilepsy, regardless of sex and age.
- The clearance of LTG was lower in postmenopausal women compared to younger women (22% lower) and older men (9% lower).
- There were notable increases in LTG clearance with co‐administration of inducers or the presence of smoking and decreases in the presence of inhibiting medications.

## 1 INTRODUCTION

Lamotrigine (LTG) is a widely prescribed antiseizure medication (ASM) for women of childbearing age and one of the most preferred ASMs in older adults.^1^, ^2^, ^3^, ^4^, ^5^, ^6^, ^7^ In postmenopausal women with epilepsy, ~40% report a worsening of their seizures, whereas 27% experience an improvement with menopause.^8^ Moreover, postmenopausal women taking ASMs face an increased risk of fractures, osteoporosis, and osteomalacia.^9^ Physiological changes—including hormone level fluctuations, increases in body fat, and increases in gastrointestinal transit times—can further impact ASM pharmacokinetics and need to be taken into account for optimum seizure control.^10^ In addition, women with hormone‐sensitive seizures may experience a change in seizure frequency due to reduced estrogen concentrations during and after menopause.^9^

LTG is metabolized primarily by the uridine‐glucuronosyltransferase (UGT) enzyme system (90%–95%). Approximately 94% of an oral LTG dose is recovered in the urine, 90% of which consists of glucuronide metabolites with the remainder being recovered unchanged.^11^ UGT1A4 and UGT2B10 are the enzymes mainly responsible for the conversion of LTG to the LTG‐N2‐glucuronide metabolite.^12^, ^13^, ^14^, ^15^ In adults taking monotherapy, LTG has a comparatively long elimination half‐life of 23–37 h^11^, ^16^, ^17^, ^18^, ^19^; however, concomitant administration of particular ASMs can change the half‐life of LTG. Enzyme‐inducing ASMs, including phenytoin, carbamazepine, primidone, and phenobarbital, decrease LTG's half‐life to 12.6–14.4 h.^20^ In contrast, the half‐life of LTG increases to 48.3–70.3 h when co‐administered with enzyme‐inhibiting ASMs such as valproate.^20^ Anticipating changes in pharmacokinetics and drug interactions is crucial for maintaining effective seizure control.

Estrogen (17β‐estradiol) is thought to upregulate UGT1A4 activity leading to increases in LTG clearance.^21^ Studies indicate that LTG clearance increases when used with estrogen‐based contraceptives.^22^, ^23^, ^24^ As estradiol (the main estrogen in fertile women) decreases after menopause, LTG apparent clearance (CL/F) may decrease compared to premenopausal levels. A decrease in LTG clearance can lead to a prolonged half‐life as well as increase the exposure of LTG, which may necessitate a dose reduction to avoid toxicity.

There is limited information on treating postmenopausal women with epilepsy. Currently, LTG is considered one of the better tolerated drugs for seizure management in the older adult population, demonstrating higher retention rates and the highest 12‐month seizure freedom rate.^2^, ^25^ However, older patients exhibit an ~27% decrease in LTG clearance compared to young patients (18‐ to 48‐year‐olds).^5^ Covariates such as weight, blood urea nitrogen/serum creatinine ratio, and co‐administration of phenytoin have been shown to have an effect on LTG clearance in older adults.^6^ Although women experience significant physiological changes that can affect LTG pharmacokinetics, there are no consistent guidelines for women who transition into this later stage of life. The aims of this study were to compare LTG prescription rates across different age groups of women and men and characterize LTG clearance variations by age and sex.

## 2 MATERIALS AND METHODS

### 2.1 Study participants

Individuals 18 years of age or older who were prescribed ASMs and whose electronic health records were collected across clinics at Fairview and University of Minnesota Physicians locations during the period from January 1, 2015 to December 31, 2021 were included in the study. Individuals were excluded if sex information was not available. The overall trend of LTG prescription among patients taking ASMs was determined. In this study, women are defined as individuals who identified as “female” in the sex information section within the database. Due to the lack of consistent menopause indicators in clinic records, age was used as a surrogate marker for menopausal status in women prescribed LTG. Women 60 of age and older were categorized as postmenopausal and those ≥18 but <60 as younger adults. The study was approved by the University of Minnesota Institutional Review Board.

### 2.2 Descriptive analysis

The master dataset included information about patients prescribed ASMs along with the International Classification of Diseases, 10th and 9th Revision (ICD‐10 and ICD‐9) diagnostic codes associated with their regimens. Patients with epilepsy were identified using ICD‐10 and ICD‐9 diagnostic codes that start with 345, 649, or G40 along with specific codes 780.33, Z81.0, R56.1, and F44.5. The LTG prescription rate, expressed as a percentage, was calculated by dividing the number of patients prescribed LTG by the total number of individuals prescribed any ASM. Prescription rates of LTG in postmenopausal women were compared to younger women and older men by individual year using a two‐proportion test. Significance was determined by *p* < .05.

Given that LTG is prescribed primarily as an ASM, our objective was to compare its prescription rates in both epilepsy and non‐epilepsy patients. Among patients prescribed LTG, supplementary analysis was done to determine the rate of monotherapy and polytherapy and to investigate potential variations in dosing regimens between individuals with and without epilepsy. An individual was considered to be on polytherapy if they were taking a second ASM in addition to LTG at any point during the given year.

### 2.3 Pharmacokinetic and statistical analysis

For pharmacokinetic analysis, additional inclusion criteria were applied: (1) diagnosed with epilepsy, (2) availability of at least one LTG blood concentration between 2015 and 2021, and (3) at steady‐state LTG dosing (defined as taking LTG at a constant dose for two or more weeks prior to sample collection). For the purpose of visual representation, patients were categorized into 5‐year age groups starting with 18‐ to 20‐year‐olds to avoid the pubertal phase. In addition, to account for the potential occurrence of menopause in women younger than age 60, a 5‐year age span was incorporated around the cutoff age. This approach was intended to capture variations in menopause onset and its potential effects on outcomes, ensuring that women who may have entered menopause slightly earlier or later than the cutoff age were still appropriately represented in the analysis. The oldest group consisted of patients 80 years or older. Because criteria included LTG concentrations reflective of steady‐state dosing, apparent oral clearance (CL/F) of LTG, which was the outcome of interest, was defined as

$$\begin{array}{cc} \mathrm{CL}/F & =\mathrm{LTG}\;\text{total daily dose}\;\left(\mathrm{mg}/\mathrm{day}\right)\\ & /\mathrm{LTG}\;\text{plasma concentration}\;\left(\mathrm{mg}/L\right) \end{array}$$

Owing to the log‐normal distribution of the outcome, the data were modeled using a linear mixed modeling approach with a log transformation of the outcome. Age group and sex were the primary predictors of interest. Concentrations below the lower limit of detection, identified by the value “<.45” in the database, were excluded, whereas concentrations below the lower limit of quantitation, indicated by the value “<.9” were assigned a value of .45 to obtain a conservative estimate of the true concentration. Clinically relevant variables were systematically tested as covariates for incorporation into the model. The model was adjusted for the following: the presence of UGT1A4 enzyme inducers and inhibitors as a part of the regimen, smoking status, and weight. A patient was classified to be taking an enzyme inducer if receiving phenytoin, carbamazepine, eslicarbazepine, primidone, phenobarbital, or oxcarbazepine, and to be taking an enzyme inhibitor if receiving valproic acid along with LTG in their therapeutic regimen. Patients were classified as tobacco smokers if their response to the tobacco usage field was affirmative (“yes”). The model was parameterized as:

$$\begin{array}{cc} \log\;Y_{\mathrm{ij}} & =\left(\beta_{0}+b_{i}\right)+\beta_{1}\times \mathrm{Age}\;{\text{Group}}_{\mathrm{ij}}+\beta_{2}\times {\mathrm{Sex}}_{i}\\ & +\beta_{3}\times {\text{Inhibitor}}_{\mathrm{ij}}+\beta_{4}\times {\text{Inducer}}_{\mathrm{ij}}+\beta_{5}\times {\text{Smoking Status}}_{\mathrm{ij}}\\ & +\beta_{6}\times {\text{Weight}}_{\mathrm{ij}}+\beta_{7}\times \mathrm{Age}\;{\text{Group}}_{\mathrm{ij}}\times {\mathrm{Sex}}_{i}+\varepsilon_{\mathrm{ij}} \end{array}$$

where *Y*_*ij*_ was the *i*th participant's LTG clearance at time *j*, *β*_0_ the intercept with participant‐specific random intercept *b*_*i*_, *β*_1_ the coefficient for age‐group indicator variable (0 for participants ≥60 years old and 1 for participants >18 but <60), *β*_2_ the coefficient for sex (0 for female and 1 for male), *β*_3_ the coefficient for inhibitor use (0 for absent and 1 for present), *β*_4_ the coefficient for inducer use (0 for absent and 1 for present), *β*_5_ the coefficient for smoking status (0 for no smoking and 1 for smoking), *β*_6_ the coefficient for the continuous covariate weight, *β*_7_ the coefficient for the age group and sex interaction, *b*_*i*_ normally distributed random effect with a mean of 0 and variability of *σ* ^2^, and ε_*ij*_ the residual error for *i*th participant at time *j*. Profile 95% confidence intervals (CIs) were calculated for relevant contrasts using the confit() command from the stats package in R. All analyses were performed in R, version 4.2.3 (R Foundation for Statistical Computing) using the packages nlme, stats, and multcomp.

## 3 RESULTS

### 3.1 Study participants

**FIGURE 1:**
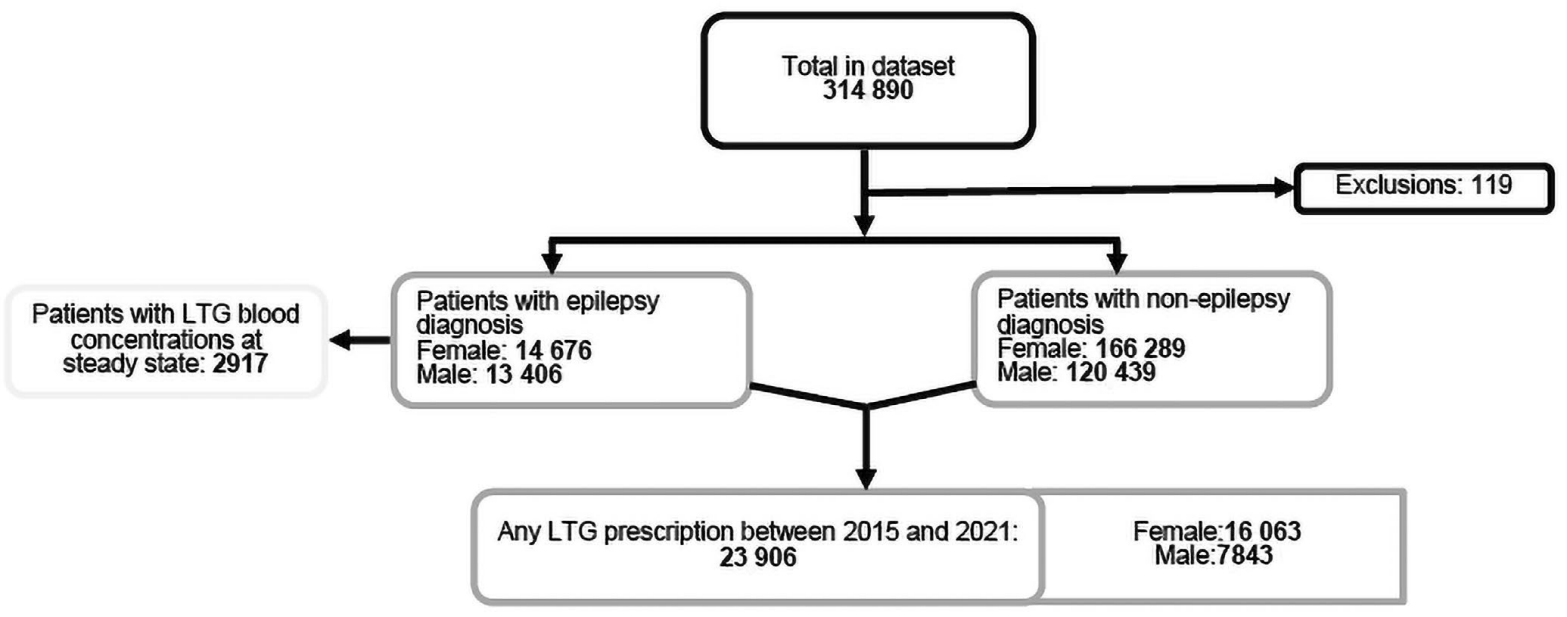
Flowchart depicting patient numbers in the analysis.

A total of 314 890 individuals (Figure 1) were prescribed an ASM between 2015 and 2021, with 23 906 individuals being prescribed LTG at least once (as monotherapy or polytherapy). Of these, 119 individuals were excluded due to missing sex. Major diagnoses for LTG prescription included epilepsy, anxiety, bipolar disorder, and depressive disorders. The total number of patients who were prescribed ASMs increased from 65 957 in 2015 (4995 received LTG) to 240 961 in 2021 (18 857 received LTG). The percentage of individuals receiving LTG as monotherapy remained constant with 50.3% (2513 of 4995) in 2015 compared to 50.2% (9463 of 18 857) in 2021.

### 3.2 Descriptive analysis: Adult women (age group effect)

**FIGURE 2:**
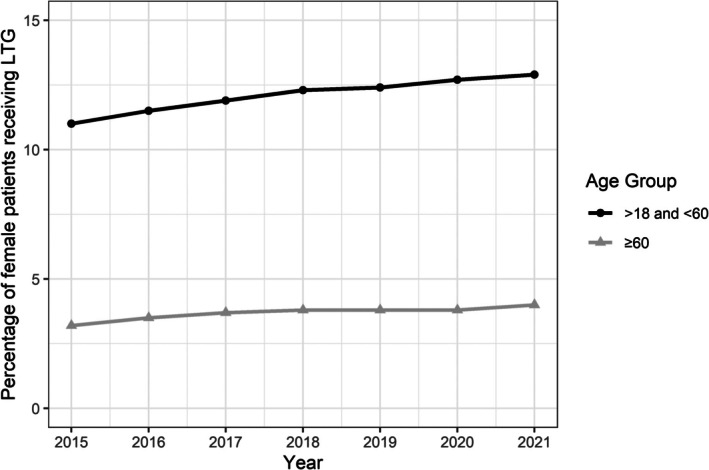
Lamotrigine prescription rates among women receiving antiseizure medications.

The total number of adult women prescribed ASMs increased from 39 479 in 2015 to 139 902 in 2021. There was a gradual increase in the proportion of women receiving LTG among women receiving ASMs from 2015 to 2021, irrespective of their age group. In postmenopausal women, LTG prescription rate increased from 3.2% in 2015 to 4% in 2021. Among adult women, the proportion of postmenopausal patients (≥60 years) receiving LTG was significantly (*p* < .001) lower than that of younger patients (≥18 but <60) across all years (Figure 2).

### 3.3 Descriptive analysis: Older adults (sex effect)

**FIGURE 3:**
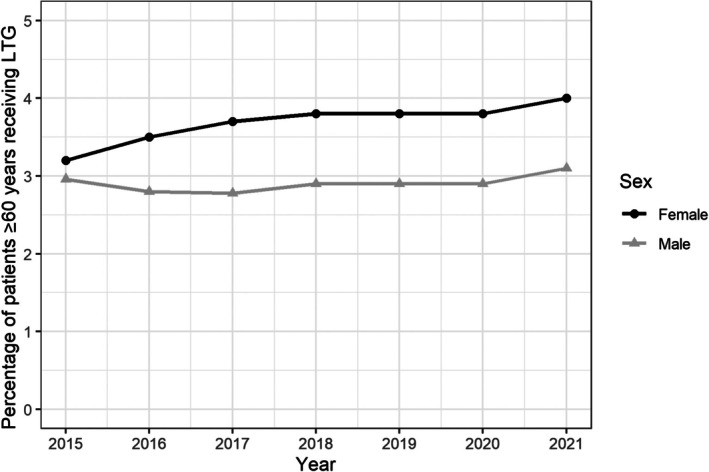
Lamotrigine prescription rates among patients receiving antiseizure medications in adults 60 years of age or older.

The total number of older adults who were prescribed ASMs increased steadily from 24 279 in 2015 to 106 795 in 2021. Over the study period there was a gradual increase observed in the proportion of older adults receiving LTG among patients receiving ASMs irrespective of sex. In patients ≥60 years of age, the proportion of women receiving LTG was significantly (*p* < .001) higher than men for all years except 2015 (Figure 3).

### 3.4 Descriptive analysis: Epilepsy vs non‐epilepsy diagnosis

**FIGURE 4:**
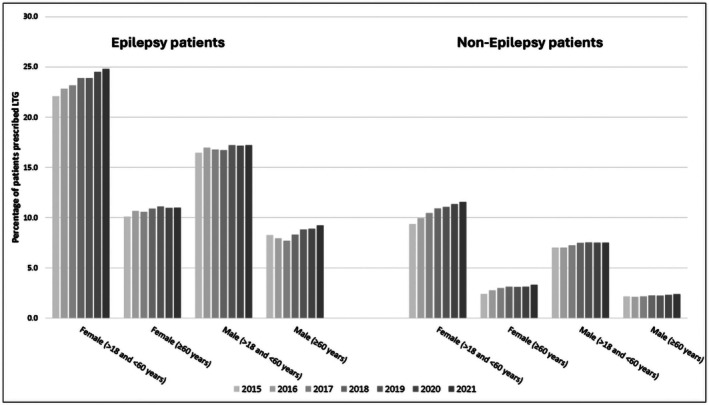
Prescription rates of lamotrigine among patients receiving anti‐seizure medications in patients with and without epilepsy.

The use of LTG as an ASM was much greater than its use for non‐epilepsy indications (Figure 4 and Table S1). A higher proportion of younger adults were prescribed LTG compared to older adults. For given age groups, women were prescribed LTG more commonly than men. LTG was prescribed less often as monotherapy in patients with epilepsy irrespective of sex and age (Figure S1 and Table S2). In patients with epilepsy, a decline in the monotherapy rates was observed from 2015 to 2021. The prescription patterns for LTG monotherapy varied between patients with and without epilepsy. Among non‐epilepsy patients, younger men had the highest LTG monotherapy prescription rates, whereas in the epilepsy group, younger men exhibited the lowest LTG monotherapy prescription rates. Postmenopausal women with conditions other than epilepsy who were taking LTG had higher monotherapy prescription rates than those with epilepsy.

### 3.5 Pharmacokinetic and statistical analysis

**FIGURE 5:**
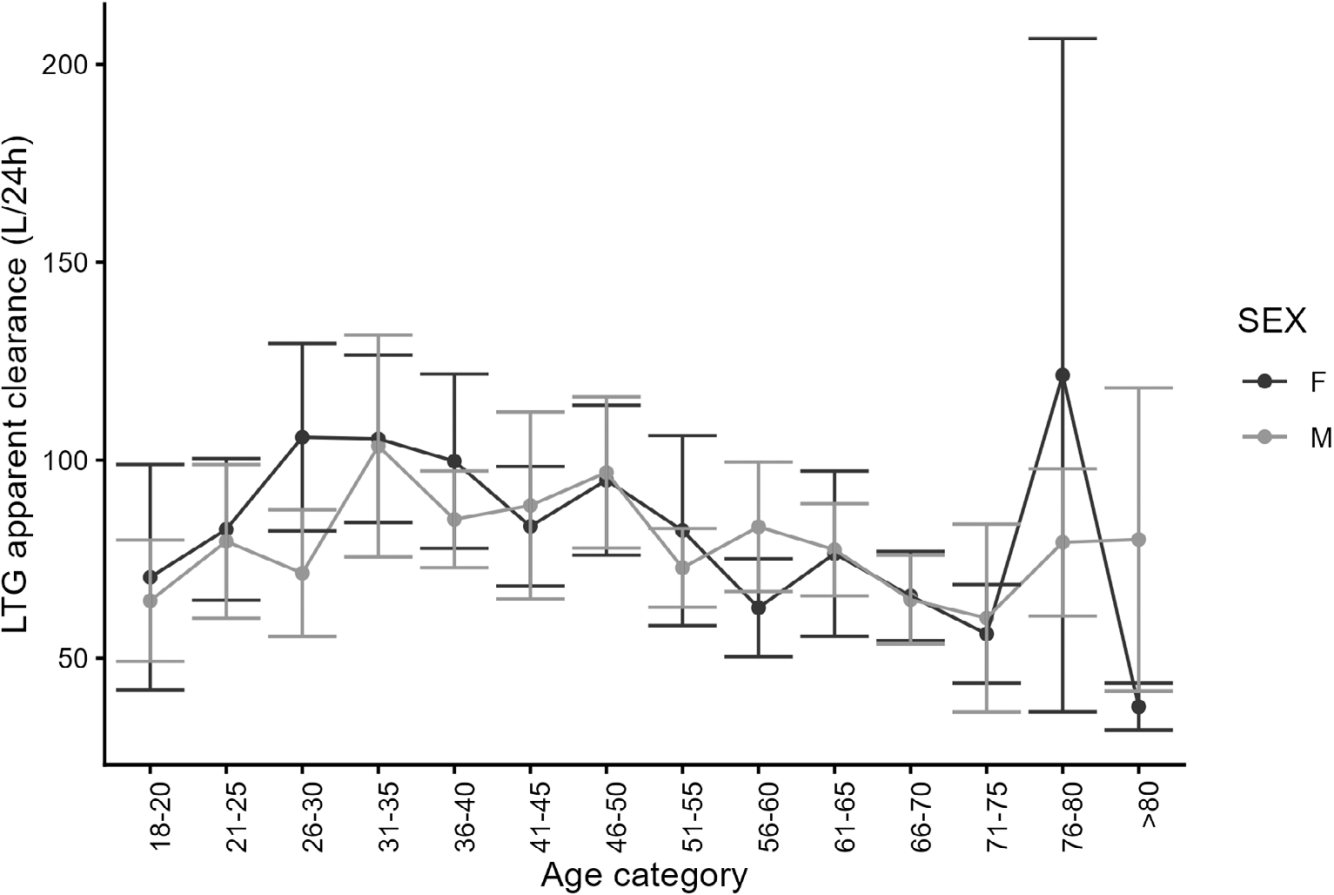
Mean lamotrigine apparent clearance with 95% confidence intervals by age category and sex.

A total of 1227 patients met the additional inclusion criteria for the pharmacokinetic analyses with 2917 concentration values available. The median number of concentrations available per individual in our study was 2 with a range of 1–21. The demographic characteristics of patients are described in Table 1. Figure 5 shows the CL/F for LTG by age and sex. As seen in Figure 5, LTG CL/F declined in the age groups between 61 and 75 years and was similar in men and women. In women 56–60 years of age, the mean (standard deviation [SD]) LTG apparent clearance was 62.1 (69.3) L/day, lower than men of the same age group at 86.8 (97.2) L/day.

**TABLE 1:** Demographic characteristics of patients.

Characteristic	Older women	Younger women	Older men	Younger men
Number of patients, *N* (%)	135 (11.0)	590 (48.1)	90 (7.3)	412 (33.6)
Number of concentrations	365	1293	231	1028
Age, Median (Q1–Q3)	67.1 (63.9–71.8)	38.1 (29.4–48.1)	65.8 (62.1–71.3)	40.2 (30.8–51.3)
Dose (mg), Median (Q1–Q3)	350 (200–550)	400 (250–600)	400 (300–550)	400 (300–600)
LTG concentration (mg/L), Median (Q1–Q3)	7.8 (4.8–11.3)	7.1 (4.3–10.7)	6.3 (4.3–9.8)	7.5 (4.6–11.1)
LTG apparent clearance (L/day), Median (Q1–Q3)	44 (32–68)	56 (38–89)	58 (42–81)	60 (38–89)
Race, *N*				
American Indian or Alaska native	1	10	0	9
Asian	3	13	0	3
Black or African American	6	38	2	32
Native Hawaiian or Other Pacific islander	1	0	0	1
White	121	490	84	343
American Indian or Alaska Native, White	0	2	0	1
Asian, Black or African American	0	1	0	0
Asian, White	0	3	0	2
Black or African American, White	0	1	0	3
NULL	3	32	4	18
Ethnicity, No.				
Hispanic or Latino	1	5	1	1
Non‐Hispanic or Latino	58	296	39	197
NULL	76	289	50	214
Smoking status, *N*				
No	118	475	77	324
Yes	16	113	12	85
NA	1	2	1	3
Co‐administration of Inducer, *N*				
No	108	510	69	312
Yes	27	80	21	100
Co‐administration of Inhibitor, *N*				
No	128	543	83	333
Yes	7	47	7	79

Abbreviations: *N*, number of individuals; Q1: first quartile; Q3, third quartile.

The results from the linear mixed‐models analysis are presented in Table S3. Younger women with epilepsy had a 22% higher (95% CI: 10.1%–35.3% higher) geometric mean clearance of LTG in comparison to older women with epilepsy (*p* < .001). In terms of older adults with epilepsy, older men had a 9% higher geometric mean clearance (95% CI: 5.5% lower–26.4% higher) of LTG in comparison to older women with epilepsy (*p* = .20). Among those with the same demographics, co‐administration of an inducer increased the geometric mean clearance by ~49% (95% CI: 39.3%–61.4%; *p* < .001), whereas co‐administration of an inhibitor reduced geometric mean clearance by ~51% (95% CI: 46.3%–55.5%; *p* < .001). In addition, those who smoked had an 11% higher LTG geometric mean clearance (95% CI: 2.3%–19.9%; *p* = .01). For every 10‐pound increase in the patient's weight, there was a 2.6% increase (95% CI: 2.0%–3.3%; *p* < .001) in geometric mean clearance.

## 4 DISCUSSION

Menopause is defined as permanent cessation of menstruation for 12 consecutive months, resulting in loss of ovarian follicle development subsequently resulting primarily in lower estrogen levels.^26^ In addition, aging includes physiologic changes that affect many organ systems, such as the renal, endocrine, cardiovascular, and gastrointestinal systems. These changes can significantly impact drug pharmacokinetics and necessitate dose adjustments. For instance, aging is associated with a decrease in the number of renal glomeruli causing a concomitant age‐related decrease in creatinine clearance, which can prolong the half‐life of certain medications in an older individual.

When considering the utilization of LTG treatment in an older individual, it is imperative to consider the influence of age‐related alterations in organ function and hormonal dynamics, some of which can significantly affect the pharmacokinetics of drugs subsequently leading to varied clinical implications.^27^ LTG is first‐line treatment for patients with focal epilepsy and bipolar 1 depression. It is also effective as add‐on therapy for generalized epilepsy.^20^ In older women, LTG could potentially be beneficial in managing new‐onset epilepsy, which can be more common in older adults. Its mood‐stabilizing properties have the potential to be useful in managing mood disorders that could arise or worsen with age. However, although LTG can have potential benefits, it may interact with other medications and as older adults often have multiple comorbidities requiring polypharmacy, the risk of drug–drug interaction or central nervous system effects can be heightened.^28^, ^29^ Such interactions may reduce LTG efficacy or increase its blood concentrations, potentially leading to toxicity.

Past studies show a rising trend in LTG use among older adults. LTG is more tolerable and effective than carbamazepine in older adults with newly diagnosed epilepsy.^2^, ^3^, ^30^, ^31^ Older adults may exhibit a reduced probability of being prescribed LTG compared to younger adults, since it is a comparatively newer ASM, approved for use in the United States in 1994. In a Brazilian tertiary sector, there was an increase in the prescription of LTG among older adults from 5.5% in 2009–2010 to 33.6% from 2015–2019.^32^ However, in regions of Finland from 2000 to 2013, older ASMs such as carbamazepine and valproic acid were the choice of drugs prescribed to this population.^33^, ^34^ Despite the documented benefits of LTG, our findings reveal a lower prescription rate for postmenopausal women compared to younger women and a higher rate compared to older men, which is similar to previous studies. We suspect that the lower utilization of LTG in postmenopausal women may be due to the possibility of drug–drug interactions and interplay of different hormones that may affect the blood concentration of LTG.^35^ It is also possible that older adults may have been prescribed other ASMs approved prior to LTG during their younger years, and subsequently are maintained on these treatments.

The typical duration of the perimenopausal phase spans ~5 years, with women reaching menopause at an average age of 50.7 years. This timeframe encompasses the age range for 95% of women, ranging from 44 to 56 years.^36^ Our study observed a decline in the apparent LTG CL in the age between 51 and 60 years. In addition, a striking difference in the apparent LTG CL was observed between men and women belonging to the age group 56–60. The findings of our study align with those reported by Tomson and colleagues, who observed a decline in apparent LTG clearance in women 51–55 years of age.^37^

Several studies report an age effect in LTG pharmacokinetics. LTG pharmacokinetics in a study of 16 younger (ages 18–48 years) and 12 older patients (ages 63–87 years) with epilepsy show LTG clearance being 27.2% lower in older patients compared to younger patients.^5^ Similar clearance changes were observed in 686 adult outpatients, where LTG (monotherapy and polytherapy) CL/F was compared between younger (age 16–36) and older patients (55–92).^38^ Median LTG CL/F of older adults taking LTG in monotherapy was ~22% lower compared to younger adults. Differences in LTG pharmacokinetics specific to post‐menopausal women is not consistent. Two studies conducted by Wegner found mixed results, with one showing an increase in LTG CL/F in postmenopausal women, which was not confirmed from the other study; however, the study that showed an increase in postmenopausal women had a small sample size.^39^, ^40^ Our study revealed a significant difference in LTG CL/F among different age groups in adult women with epilepsy. Specifically, our findings indicated that younger women with epilepsy exhibited a 22% higher mean geometric LTG clearance compared to their older counterparts within the study cohort. Our study identified a 9% higher LTG CL in older men compared to postmenopausal women with epilepsy, although the difference did not reach statistical significance. Smoking as shown in the previous studies was associated with an increase in the CL/F of LTG.^40^, ^41^ Co‐administration of an inducer or inhibitor was an additional factor shown to affect the CL/F of LTG and thus should be taken into consideration when finding the optimum dose for patients prescribed LTG.^41^

There were several limitations of our study. Due to lack of a specific indicator for postmenopausal status such as serum hormone concentrations (luteinizing hormone, follicle stimulating hormone, estrogen, and progesterone), age ≥60 years was used as a surrogate marker for menopause. The selected cutoff age for menopause was chosen conservatively to encompass all women in the group. To account for the potential occurrence of menopause in women younger than 60 years, a 5‐year age span was incorporated for comparative analysis. In certain cases when medications were started in the previous year and stopped in any subsequent year(s), patients would still be considered taking that particular medication for the entire year. Similarly, if patients switched to a different medication during a particular year, they would be classified as taking polytherapy rather than monotherapy, since the entire year as a unit was considered for analysis purposes. As it was assumed that medication lists are promptly and accurately updated by the physician, there can be errors due to delayed removal of medications or dosing changes in clinical notes but that are not reflected in prescriptions. If the patient changed institutions, their medication list could have inaccuracies. If the patient had multiple diagnosis codes associated with their prescription and one of them was related to epilepsy, they were assumed to be receiving LTG for their epilepsy treatment. Due to the absence of information on precise timing of LTG doses by the patient, the concentrations of the drug may be variable due to overall fluctuation in a day and dosing interval; however, LTG has a long half‐life compared to dosing interval, and fluctuation over a day should be minimal. A small percentage of data (<1%) was excluded due to missing sex information for participants. Since this is a retrospective database study, we did not have predefined sampling time points. Therefore, we included all patients with at least one concentration value in our model‐based analysis. Given the limited number of cases with missing sex data, although bias is expected, it is likely minimal and does not impact the overall conclusions of the study.

## 5 CONCLUSIONS

The use of LTG is lower in postmenopausal women compared to younger women but higher than in older men. Postmenopausal women were prescribed LTG as monotherapy to a lesser extent than older men and younger women. Patients with epilepsy have a higher chance of receiving LTG as polytherapy in comparison to non‐epilepsy LTG‐treated patients. Our study suggests a potential decline in LTG clearance in the perimenopausal period, although the precise reason underlying this requires further investigation. Postmenopausal women may require a dose reduction in their therapy to mitigate potential dose‐related adverse effects. Based on our study, women taking LTG should be closely monitored using therapeutic drug monitoring as they transition into perimenopause or menopause, as timing of the changes will be different for each individual.

## AUTHOR CONTRIBUTIONS

Yuhan Long was responsible for data cleaning and management. Charul Avachat designed the research, conducted data analysis, and drafting of manuscript and revisions. Angela K. Birnbaum and Sima I. Patel were involved in design and critical review and revision of manuscript. Ashley Petersen helped guide the biostatistical analysis of the data. All authors have read and approved the final manuscript.

## CONFLICT OF INTEREST STATEMENT

Charul Avachat: No conflicts. Yuhan Long: No conflicts. Ashley Peterson: No conflicts. Angela K. Birnbaum: Dr. Birnbaum has received support from the National Institutes of Health, Randy Shaver Cancer and Research Foundation, Vireo Health LLC, and UCB Pharma. Sima I. Patel: No conflicts. We confirm that we have read the Journal's position on issues involved in ethical publication and affirm that this report is consistent with those guidelines.

## Supporting information


Figure S1

Tables S1–S3

## Data Availability

Data sharing is not applicable to this article as no new data were created or analyzed in this study.
