# Supplementary figures and images for: Age‐ and sex‐associated variability in lamotrigine prescription patterns and clearance
Source: Epilepsia. 2026 Feb 2;67(3):1256–66. doi: 10.1111/epi.70028 (PMC13007825; doi:10.1111/epi.70028)

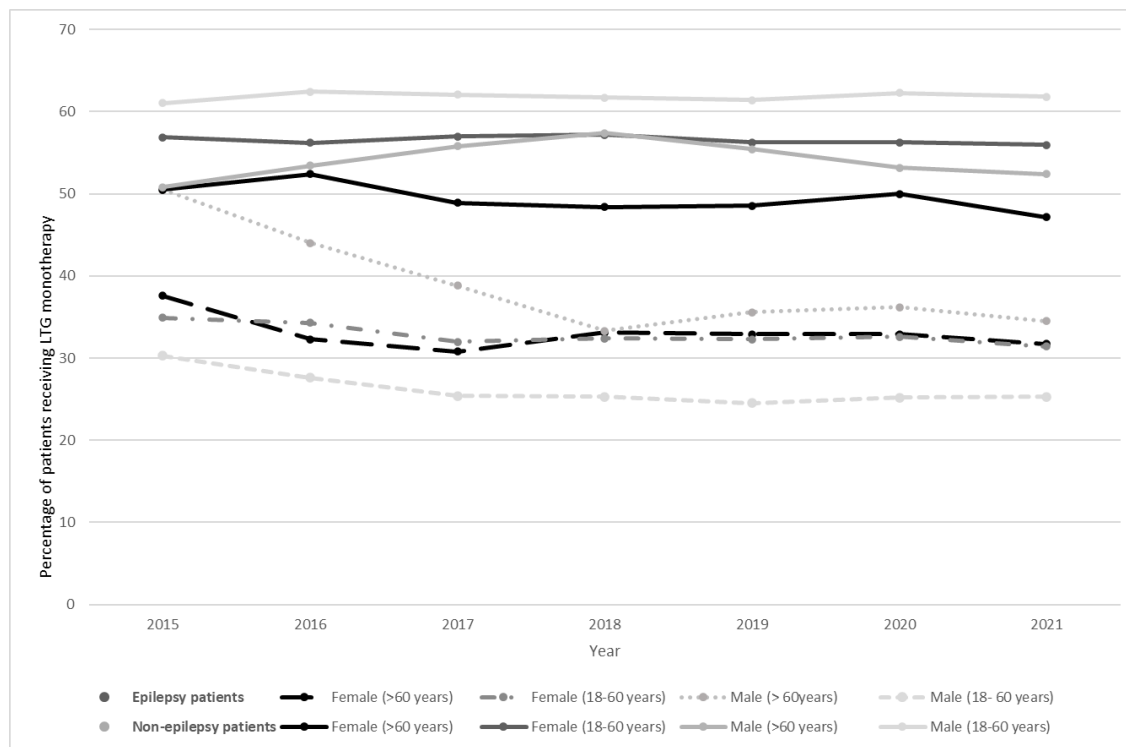

Supplement: Supplementary file 1 — Figure S1 [file EPI-67-1256-s002.pdf]
