# Supplementary material for: Age‐ and sex‐associated variability in lamotrigine prescription patterns and clearance
Source: Epilepsia. 2026 Feb 2;67(3):1256–66. doi: 10.1111/epi.70028 (PMC13007825; doi:10.1111/epi.70028)
Supplement: Supplementary file 2 — Tables S1–S3 [file EPI-67-1256-s001.docx]

**Table S1.** Percentage of patients prescribed lamotrigine among patients receiving ASMs: epilepsy vs non- epilepsy patients

| **Year** | **2015** |  | **2016** | **2017** | **2018** | **2019** | **2020** | **2021** |  |
| --- | --- | --- | --- | --- | --- | --- | --- | --- | --- |
| Epilepsy patients | | | | | | | | | |
| Female (18- 60 years) | 22.1% |  | 22.8% | 23.2% | 23.9% | 23.9% | 24.5% | 24.8% |  |
| Female (>60 years) | 10.1% |  | 10.7% | 10.6% | 10.9% | 11.1% | 11.0% | 11.0% |  |
| Male (18- 60 years) | 16.5% |  | 17.0% | 16.8% | 16.7% | 17.2% | 17.2% | 17.2% |  |
| Male (>60 years) | 8.3% |  | 7.9% | 7.7% | 8.3% | 8.8% | 8.9% | 9.2% |  |
| Non-epilepsy patients | | | | | | | | | |
| Female (18- 60 years) | 9.4% |  | 10.0% | 10.5% | 10.9% | 11.1% | 11.4% | 11.6% |  |
| Female (>60 years) | 2.4% |  | 2.8% | 3.0% | 3.1% | 3.1% | 3.1% | 3.3% |  |
| Male (18-60 years) | 7.0% |  | 7.0% | 7.2% | 7.5% | 7.5% | 7.5% | 7.5% |  |
| Male (>60 years) | 2.2% |  | 2.1% | 2.2% | 2.3% | 2.2% | 2.3% | 2.4% |  |

**Table S2.** Percentage of patients prescribed lamotrigine monotherapy: epilepsy vs non-epilepsy patients

| **Year** | **2015** | **2016** | **2017** | **2018** | **2019** | **2020** | **2021** |  |
| --- | --- | --- | --- | --- | --- | --- | --- | --- |
| Epilepsy patients | | | | | | | | |
| Female (18-60 years) | 34.9% | 34.3% | 32% | 32.4% | 32.3% | 32.6% | 31.4% |  |
| Female (>60 years) | 37.6% | 32.3% | 30.8% | 33.1% | 32.9% | 32.9% | 31.7% |  |
| Male (18- 60 years) | 30.3% | 27.6% | 25.4% | 25.3% | 24.5% | 25.2% | 25.3% |  |
| Male (>60 years) | 50.5% | 44% | 38.8% | 33.3% | 35.6% | 36.2% | 34.5% |  |
| Non-epilepsy patients | | | | | | | | |
| Female (18-60 years) | 56.9% | 56.2% | 57.0% | 57.2% | 56.3% | 56.3% | 56.0% |  |
| Female (>60 years) | 50.5% | 52.4% | 48.9% | 48.4% | 48.5% | 50.0% | 47.1% |  |
| Male (18-60 years) | 61.1% | 62.4% | 62.1% | 61.7% | 61.4% | 62.3% | 61.8% |  |
| Male (>60 years) | 50.8% | 53.4% | 55.8% | 57.4% | 55.4% | 53.2% | 52.4% |  |

**Table S3** Results from the linear mixed model examining the effect of age and sex on apparent clearance of lamotrigine

| **Coefficient** | **Estimate** | **95% CI** | **P-value** |
| --- | --- | --- | --- |
| Intercept (β0) | 30.77 | 26.670- 35.490 | <0.001 |
| Age group: Younger (β1) | 1.22 | 1.100-1.352 | <0.001 |
| Sex: Male (β2) | 1.09 | 0.945-1.264 | 0.20 |
| Inhibitor (β3) | 0.49 | 0.445-0.537 | <0.001 |
| Inducer (β4) | 1.49 | 1.393-1.614 | <0.001 |
| Smoking Status (β5) | 1.11 | 1.023-1.199 | 0.01 |
| Weight:10lb increase (β6) | 1.026 | 1.020-1.033 | <0.001 |
| Age group * Sex (β7) | 0.87 | 0.745-1.024 | 0.097 |
